# When Sugar-Coated Words Taste Dry: The Relationship between Gender, Anxiety, and Response to Irony

**DOI:** 10.3389/fpsyg.2017.02215

**Published:** 2017-12-19

**Authors:** Anna Milanowicz, Adam Tarnowski, Barbara Bokus

**Affiliations:** Faculty of Psychology, University of Warsaw, Warsaw, Poland

**Keywords:** irony, gender bias, anxiety, blame by praise, praise by blame, humor, malice

## Abstract

This article approaches the question of mocking compliments and ironic praise from an interactional gender perspective. A statement such as “You're a real genius!” could easily be interpreted as a literal compliment, as playful humor or as an offensive insult. We investigate this thin line in the use of irony among adult men and women. The research introduces an interactional approach to irony, through the lens of gender stereotype bias. The main question concerns the impact of individual differences and gender effect on the perception and production of ironic comments. Irony Processing Task (IPT), developed by Milanowicz (2016), was applied in order to study the production and perception of ironic criticism and ironic praise in adult males and females. It is a rare case of a study measuring the ability to create irony because, unlike most of known irony research, it is not a multiple choice test where participants are given the response options. The IPT was also used to assess the asymmetry of affect (humor vs. malice) and impact of gender effect in the perception of ironic comments. Results are analyzed in relation to the State-Trait Anxiety Inventory (STAI) scores. The findings reveal the interactional relationship between gender and response to irony. Male responses were consistently more ironic than female's, across all experimental conditions, and female responses varied more. Both, men and women used more irony in response to male ironic criticism but female ironic praise. Anxiety proved to be a moderate predictor of irony comprehension and willingness to use irony. Data, collected in control and two gender stereotype activation conditions, also corroborates the assumption that the detection of compliments and the detection of criticism can be moderated by the attitude activation effect. The results are interpreted within the framework of linguistic intergroup bias (LIB) and natural selection strategies.

## Introduction

“L'humour est une disposition d'esprit qui fait qu'on exprime avec gravité des choses frivoles et avec légèreté des choses sérieuses.“

Alfred Capus

Irony is wordplay, a figure of speech that flouts the maxim of quality, requiring information provided in conversation to be truthful (Grice, 1975). However, it implies the contradiction of what is literally expressed. It is characterized by opposition and substitution between two levels of meaning. It is an Aristotelian blame-by-praise figure, criticism which sounds like a compliment, where in fact, what the speaker literally says should be taken to mean “something else,” conveniently assumed to be the exact or relative opposite of what is said. Irony can be humorous and humor can be ironic—these two concepts may overlap but are not tantamount.

Every day we feed ourselves with words and ideas, we process them and, in doing so, we often refer to stereotypes. We cook facts, we simmer with resentment, and we cool down. We find certain opinions indigestible, some humor dry, and some comments sharp. Words make us feel sick or satisfied. Most importantly, however, we do not always find the same platter equally tasty. What one person finds savory and pungent might seem quite insipid and bland to another.

We believe that this is exactly what happens with language. Some enjoy refinement and undertones while others appreciate simplicity and directness. Irony can evoke laughter, but its humorous potential might also not be recognized and taken instead as stinging and harsh. But who likes what? What are the flavors of irony? In our attempt to understand the varied research results on social functions of irony (Kreuz et al., 1991; Dews et al., 1995; Pexman and Olineck, 2002) as well as its contradictory nature itself (Grice, 1975; Giora, 1995; Gibbs and Colston, 2007) we decided to combine the so far-disclosed qualities and components and concoct a new recipe for its nature.

To this end, we designed four scenarios gathering men and women's spontaneous responses to the same ironic criticism (*blame by praise*, BbP) or ironic compliment (*praise by blame*, PbB) voiced by either ingroup (same sex) or outgroup (opposite sex) interaction partner in control and two gender stereotype activation conditions. We decided to use BbP and PbB labels (Anolli et al., 2002) due to the fact that they seem least confusing, as compared with “critical praise,” “critical blame,” “ironic compliments,” and “ironic criticism”. As Burgers et al. (2012) rightly point out,

the terms ironic praise and ironic blame are used in two distinct ways in the irony literature. Some authors use ironic praise to refer to ironic utterances that are literally negative, such as “That's a horrible idea” (e.g., Schwoebel et al., 2000; Filipova and Astington, 2008). In contrast, other irony scholars define ironic praise in the exact opposite way, namely, by referring to ironic utterances that are literally positive, such as “That's a great idea” (e.g., Poggi et al., 2007; Poggi and D'Errico, 2010) (p. 306).

### Irony as a verbal dimension of social comparisons

In general perception, irony is a funny thing (Kreuz and Glucksberg, 1989; Roberts and Kreuz, 1994; Colston and O'Brien, 2000). Ironic remarks are viewed as more playful than literal comments (Kreuz et al., 1991; Gibbs, 2000) and people who use irony are perceived as having a sense of humor (Pexman and Olineck, 2002). Other than humor, politeness is also indicated as a communication goal of irony. Leech (1983) makes reference to his *politeness principle* and proposes an *irony principle*, where irony is seen as a way of not causing offense directly and thus preventing an open conflict. However, results of research conducted by Matthews et al. (2006) indicated that humor, but not politeness, was a significant factor in a speaker's decision to use verbal irony. According to Partington (2007), the use of irony is affiliative inasmuch as it can “bind speaker and hearer when a third party is the object of criticism, it can be used in friendly teasing or it can be used in self-deprecatory humor” (p. 1,565). While Dews et al. (1995) propose the *tinge hypothesis* and tinge function of irony, namely, muting the aggression expressed in criticism and moderating the praise communicated in a complement, Brownell et al. (1990) show that, actually, ironic criticism can be rated as “meaner” than literal criticism. Ironic comments can be perceived as “mocking” (Kreuz et al., 1991) and implying the intention of being more hurtful (Pexman and Olineck, 2002).

“The study of humor, irony, and other playful forms is plagued by definitional problems” (Attardo, 2002, p. 166) and there is no common understanding among researchers as to what irony is. From Ancient Greek, irony, ε,ρ*ωνε*íα *(eirōneía)*, means a pretended ignorance. According to Encyclopædia Britannica (https://www.britannica.com), the term irony has its roots in the Greek comic character Eiron, a clever underdog who by his wit repeatedly triumphs over the boastful character Alazon. From being a violation of code, a figure of speech that does not mean what it says, flouting the maxim of quality (Grice, 1975), through the game of pretense (Clark and Gerrig, 1984), to the sound of an echo (Sperber and Wilson, 1981, 1984; Kumon-Nakamura et al., 1995), and indirect negation (Giora, 1995), irony still means more than its literal words.

According to Dynel (2014), there is no clear distinction in the topical literature between humorous irony and non-ironic humor, and “linguistic phenomena displaying overt untruthfulness and humor may be easily mistaken for humorous irony.” (p. 621). Due to the fact that researchers of irony face an array of interfering variables, Burgers et al. (2012) propose five requirements for an ironic utterance to be qualified as ironic: *evaluatieveness, incongruence* (between the literal meaning of the irony and its co- or context), *reversal of valence* (i.e., irony with a positive literal meaning, as in “Good idea, John!” when the idea was bad or irony with a negative literal meaning, as in “Bad idea, John!” when the idea was good), *target* (irony is always aimed at somebody or something), and *relevance to the communicative situation*. Instead of five, Dynel (2014) proposes a set of two “conditions serving as an acid test for irony,” namely: (a) overt untruthfulness and (b) negative evaluation.

Given the multiple definitional problems and operational challenges resulting from lack of any specific measure of irony, it is not surprising that research on irony brings conflicting and contradictory results. However, differences in irony perception might also result from the simple fact that we differ in how we see the world, what we like about it and how we describe it; this may explain why there is systematic variance in irony detection performance (Bruntsch et al., 2016). Previous research by Milanowicz (2013) showed that that men would use irony with the aim to amuse others, to make fun, and to be perceived as funny, but women would rather use ironic comments to show their disapproval and smuggle in more anger and meanness. Seeing clearly the variability in the load of ironic comments, we agree with Jorgensen (1996) that in order to see how irony can be used as an effective tool for communication, we should look into the perceptions of ironic instances. This is exactly what we are doing here. We measure production and evaluation of irony in relation to individual differences, such as gender and anxiety.

Irony can convey meanings outside the humorous frame, unlike teasing, but is not necessarily aimed at hurting others, unlike sarcasm. Essentially, according to Dynel (2014), the difference between irony and sarcasm comes down to the overt untruthfulness typical of irony, while the difference between irony and teasing is based on the negative evaluation, not always present in the latter category of humor. As mitigating, funny, critical, or mean as irony might seem, still, the ballistic repertoire used to describe its humor and “barbs” tips the scales of verbal interaction more in favor of the battlefield rather than a playground setting. In the subject matter literature, we encounter “targets” or “victims” of ironic comments, we read about “aims” of ironic remarks and “face-threatening” or “face-saving” techniques. Given that we are discussing hidden meaning, where *implicatures* must be pulled out from communication like rabbits from a hat, maybe it would be more appropriate to employ more of a wording that alludes to verbal illusion.

Also illusory for some researchers is the possibility of so-called asymmetry in irony, that is, the notion of critical (negative) irony being more frequent than praising irony (Sperber and Wilson, 1981; Clark and Gerrig, 1984; Matthews et al., 2006) While most theories approach “ironic praise” and “ironic blame” as two categories of the same genre, some researchers (Garmendia, 2010) voice their veto and deny the possibility of being ironic without criticizing —stating that the asymmetry issue is an illusion. We were quite inclined to follow that path, until our research results made us look at this asymmetry through the lens of the *linguistic intergroup bias* theory (Maass et al., 1989; Maass, 1999; Wigboldus and Douglas, 2007), which states that positive ingroup descriptions and negative outgroup descriptions are abstract and vague, while negative ingroup descriptions and positive outgroup descriptions are specific and observable. In other words, desirable behavior of ingroup members is interpreted on an abstract cognitive level, while negative behavior is interpreted on a more concrete level. This phenomenon is reversed when interpreting outgroup member behavior, which helps maintain a more positive image of one's own group.

We assume, in light of this theory, that if we accept irony as the manifestation of non-literal, indirect, and somehow abstract language, as opposed to direct and literal language, we can expect that when addressing one's own group (the ingroup, i.e., a same-sex interlocutor), irony should be used rather in a positive context (e.g., to praise), while literal language will be employed in a negative context. The opposite will be true toward anyone in the outgroup (i.e., an opposite sex interlocutor), and use of irony should then be preferred in negative contexts (e.g., to criticize). Thus, we assumed that women and men will understand irony communicated by a same-sex person differently than if communicated by an opposite-sex person, and we ventured into gender stereotype activation in the process of verbal communication.

### Irony and social comparisons based on gender

Dunin (2001) attributes the common perception of gender to stereotypes based on polar opposites: aggressive and gentle; insensitive and caring; mathematician and linguist; talkative and quiet; logical and intuitive; competitive and co-operative, pink and blue. For Dunin, people are different from one another, regardless of gender, but it is precisely “the stereotypical visions of femininity and masculinity, ingrained in our minds and cultures, that differentiate people in an extremely simplified way.” (Dunin, 2001).

It seems understandable that by attaching importance to these differences, which we are fed since birth and which we feed on every day, it is difficult not to react automatically to the “other” for which, in this paper, we use the term *outgroup member*. Gender differences in self-construal are to a large extent the product of social comparison processes (Guimond et al., 2007) and not any comparisons, but especially intergroup comparisons (Guimond et al., 2006). The conceptions of ingroup identity varying as a function of comparative context are already present in children (Sani et al., 2003). We self-categorize and categorize others in an attempt to balance our identity between individualism and a sense of belonging without risking either alienation or loss of identity (Turner et al., 1987; Guimond et al., 2006).

Recchia et al. (2010) showed that in family conversations at Canadian homes, mothers were likely to ask rhetorical questions and use ironic language in conflictual contexts, while fathers used hyperbole and understatement as frequently as rhetorical questions, and employed ironic language in both positive and conflictual contexts. Kałowski (2017) introduced audiovisual stimuli (recordings of women and men making ironic statements directed at the participant) and collected data in the form of recordings of utterances, and their analysis was consistent with previous research showing that males feel more positive about using irony (Jorgensen, 1996; Colston and Lee, 2004; Milanowicz, 2013). Self-stereotype activation yielded higher humor ratings of irony than non-irony used by male (but not female) actors in the stimulus videos. Thus, it is possible that “meaning well when using irony” is part of a male stereotype accessed by both genders in intergroup comparison conditions. Analogously, “not meaning well when using irony” might be an element of a female stereotype. These results also suggest that men and women might use irony for different reasons (Milanowicz, 2013; Milanowicz and Kałowski, 2016; Kałowski, 2017).

On a regular basis, we are involved in social networks and growing within the frames of our times and mores, we take it as natural to put labels onto things we see. We name these things, like them, hate them, use them, and talk about them, but do we understand them? Because of the pervasiveness of “name tags” and identification labels in social interactions, social inclusions and exclusions, of which gender stereotypes are also a manifestation, we decided to throw down the gauntlet and see how blue boys play “irony games” with pink girls.

We agree with Hyde (2005) that gender is not only a person variable but, most importantly, a social stimulus, whose activation, like the activation of any other stereotype, impacts an individual's perception, judgments and behavior (Bodenhausen and Lichtenstein, 1987; Devine, 1989; Bargh et al., 1992; Macrae et al., 1994). We understand that gender studies are quite controversial and raise a lot of emotions. This can also be one of the reasons why, maybe quite surprisingly, given the vastness of the phenomenon, there is not much research on irony and gender. The aim of this research is not to present any misconception of gender equalities or differences. We take the category of gender as a classification subcategory, a social cluster which might help to explain the ambiguity of cognitive meanings and verbal behavior engaged in the response to one and the same stimulus: irony.

Gibbs (2000) reported that men were more likely than women to use sarcastic irony in conversation with friends. Jorgensen (1996) examined the effect of gender on the social and emotional impact of irony and reported that men were more likely than women to perceive humor in sarcastic irony and women were more likely than men to be offended or angered by sarcastic remarks. Same results were obtained in research by Milanowicz (2013). Katz et al. (2001) have investigated whether gender, as a social category, could suggest a speaker's tendency to make ironic remarks. In the light of their data, men were perceived to be more sarcastic than women. Holtgraves (2005) found gender differences in how participants rated their own tendencies to speak sarcastically, with male participants presenting higher self-reports of use of sarcasm than female participants. However, most of the research on irony might raise the question about consistency between what people claim to be (on paper or in the laboratory condition) and how they actually behave.

Lampert (1996) suggested that the primary motive for men using conversational humor is the reduction of social vulnerability: “irony can serve the self-protective function that Lampert claims is important to men and, indeed, men's ratings suggested they were more likely to use irony in most situations” (Ivanko et al., 2004, p. 266). Holtgraves (1997) showed that men rated themselves higher than women on the production factor of the Conversational Indirectness Scale (CIS)—devised to measure individual tendencies to express and interpret meanings indirectly. Irony, as the manifestation of indirect criticism, can give the impression of politeness. “Participants with higher CIS–production scores seemed more apt to recognize this politeness function, whereas female participants tended to recognize the critical (and thus impolite) function of ironic criticisms” (Ivanko et al., 2004, p. 265). In the research by Ivanko et al. (2004), females rated ironic compliments as being more sarcastic than did the male participants. The authors explain it by greater sensitivity on the part of female participants to the negative tinge of ironic compliments (Dews et al., 1995; Pexman and Olineck, 2002) The gender differences reported in the research by Ivanko et al. (2004) replicated Jorgensen's (1996) observed differences between male and female perception of politeness in ironic comments.

Colston and Lee (2004) found that irony is considered a more male-like than female-like form of communication by both men and women, reporting that “fictional speakers of unknown gender who use verbal irony to comment about relatively negative situations are thought to most likely be male” and that “males report a greater likelihood of using verbal irony in negative situations” (Colston and Lee, 2004, p. 301). They posit that men tend to use irony more often than women because their pragmatic goals in conversations more often include expressing a critical lack of approval. Alternatively, men could be more ironic because they show a greater propensity toward risk-taking (Colston and Lee, 2004) and use of irony involves a certain risk of being misunderstood. We assume that those differences might be explained not only by the willingness to take risk, but also in terms of reluctance and fear to venture into what is unknown and/or ambiguous, of which non-literal language is a representation on the symbolic level.

### Irony and anxiety

Similarly to most research on humor, most studies on irony also focus on its positive qualities.

Ruch and Proyer (2008a,b) were the first to study gelotophobia (the fear of being laughed at) empirically as an individual differences variable that characterizes the degree to which people fear being laughed at by others. (Chłopicki et al., 2010, p. 172)

Comparatively, we believe that the application of the anxiety measure (STAI) combined with the analysis of not only funniness but also meanness in perception of verbal irony (IPT) allows for a more dimensional approach to the whole concept. It goes without saying that that *laughing at* and *laughing with* are not equipollent. Introduction of anxiety to the research on irony can be seen as a prelude to further assessment of the links between the perception of being laughed at and the motivation to laugh at others or ridicule them. Also, the Polish GEOLPH < 15>, the Polish adaptation of the Inventory for Assessing Gelotophobia by Ruch and Proyer (2008b), showed that the fear of being laughed at existed widely independently from the age or sex (Chłopicki et al., 2010). Research on irony showed differences between men and women and thus we are curious to know if these differences are related to anxiety?

Both anxiety and irony relate to emotional experience and lead to emotional responses. Irony as an unexpected and ambiguous stimulus can evoke a state of “fight or flight” alertness. It is believed that, in order to arrive at a response to such a stimulus, we instinctively refer to our cognitive schemas, personal knowledge, and emotional attitude. Attitudinal responses are evaluative, and evaluation is connected with the imputation of some degree of goodness or badness to an entity (Lewin, 1935). Valence refers to intrinsic attractiveness (positive valence) and aversiveness (negative valence) of an event, situation, object, or stimulus (Lewin, 1935; Damasio, 1994), thus, affective valuation should be viewed as an integral part of meaning.

Irony being an ambiguous stimulus can be perceived not only as a harmless joke but also as threatening. Studies on interpretive and judgment biases indicate that they are already present in children with anxiety, leading them to interpret ambiguous stimuli as threatening (Taghavi et al., 2000) and exhibit avoidant responses (Chorpita et al., 1996). Another study on individual differences in children exploring associations between verbal irony comprehension and shyness (Mewhort-Buist and Nilsen, 2013) reported that shyer children ascribed a greater degree of negative attitude to speakers who made ironic criticisms. It was also demonstrated that children higher in shyness showed less appreciation of the irony muting effect.

We thus hypothesized that higher anxiety levels in adults could also lead to defensive performance in humor appreciation of irony. However, in line with the widely accepted belief that women are more emotional (Brody and Hall, 2008), more emotionally expressive than men (Kring and Gordon, 1998), and more likely to suffer from clinical anxiety than men (Remes et al., 2016), it was believed also that this effect will be moderated by gender.

Women show a greater tendency than men to interpret utterances as figurative (Holtgraves, 1991). However, a higher level of anxiety could account for the perception of ironic (ambiguous) comments as being more threatening because it seems more unknown and so more scathing than literal criticism.

Also, gender stereotypes provide a basis for socializing boys and girls about appropriate emotional behavior, where expressing fear and sadness is acceptable for girls but not for boys (Brody, 2000; Chaplin and Aldao, 2013). This emotional double-standard associated with the stereotype serves the function of preserving the social hierarchy, where women are viewed as irrational and uncontrollable and thus dangerous, legitimizing women's subordinate rank in the power hierarchy (Lutz, 1996).

We approach gender as the set of behaviors and attitudes that characterize people of a given biological sex. In this paper we write about gender (variable) because we show the existence of different patterns of verbal behavior in men and in women. These differences can also result from acceptance of different social roles pertaining to sex.

We have also decided to give importance to gender and anxiety in irony research because women are almost twice as likely as men to experience anxiety. This gender gap might result from physiological factors but also might be related to differences between men and women in how they cope with stress (Remes et al., 2016).

Therefore, it remains important to consider how inequalities among men and women in this respect might contribute to their different approaches to irony.

Due to its ambiguous nature (uncertainty as to the real meaning and interpretation), irony can possibly be a stressful stimulus for some people. Therefore, we deem it legitimate to include anxiety as an individual variable modifying human reactions to irony.

## Materials and methods

### Ethics statement

This study was carried out in accordance with the recommendations of the Academic Ethical Review Board (Scientific Research Ethics Committee of the Faculty of Psychology, University of Warsaw). The participants provided verbal informed consent to take part in the study. Such a form of consent is customarily used in Poland in studies on adult student samples. The consent procedures were detailed in a description submitted to the institutional review board (Ethics Committee of the Faculty of Psychology, University of Warsaw), where they were granted final approval in October 2014. The participants were granted full anonymity of the data gathered for the analyses and were informed that only group results will be described.

### Participants

Participants were recruited from among students (University of Warsaw and Warsaw University of Technology) and public institution employees. They participated voluntarily in the study and returning the completed questionnaires meant their consent to take part in the study. The total sample consisted of 238 subjects (*M*_age_ = 23.92; *SD* = 8.120): 127 females, age ranged from 18 to 44 (*M*_age_ = 21.31; *SD* = 4.727) and 111 males, age ranged from 18 to 60 (*M*_age_ = 26.89; *SD* = 9.987).

### Measures

The State-Trait Anxiety Inventory (STAI, Polish adaptation, Spielberger et al., 1987)—was distributed among participants in order to verify if anxiety can be shown to predict perception of ironic funniness or ironic meanness. The STAI contains two 20-item scales measuring state and trait anxiety. All items are rated on a 4-point scale, ranging from “almost never” to “almost always.” Both anxiety scales were used in this study. The Cronbach's alpha for the state anxiety scores was 0.928, while the Cronbach's alpha for the trait anxiety scores was 0.897.

The Irony Processing Task (IPT, Milanowicz, 2016)—a self-report questionnaire was designed to stimulate production of non-literal comments and measure not only comprehension but, most importantly, the reaction to ironic comments. The task consists of six scenarios, each depicting a short context introduction with a simple cartoon and a comment. Due to the general belief that females tend to perform better on tasks requiring decoding of non-verbal information (Hall, 1984; Collignon et al., 2010), the IPT was designed in such a way as to eliminate those cues. Any indicators of a prosodic or kinetic character, as well as facial cues, were neither considered nor present. The cartoons and ironic comments were followed by dialogue balloons for participants to write down their spontaneous replies. Some of the scenarios were followed by questions about the motivations of the speakers and emotions of the recipients. Some other actions and comments were evaluated on two five-point rating scales of “humor” and “malice” as follows: 1 (*not at all*)−5 (*very*) The IPT was developed in order to see what relationship, if any, exists between gender and response to irony. The measure aims to see how irony is understood and produced in different communicative settings and how irony is used toward different communication partners. Also, we decided to use this mode (the written word) because this is the way in which we communicate nowadays in the era of digital communication, which seems to be taking over certain aspects of face-to-face interaction.

In this paper we describe the results of two IPT experimental tasks.

The first experimental task, IPT 1, involves four context scenarios and four target statements, where the participant is asked to imagine that each ironic comment is voiced directly toward him or her. Half of these comments (one expressed by female and one expressed by male) are ironic criticism, (BbP, i.e., positive literal meaning but negative true meaning, like “Genius!” when the idea made by the participant is very bad and what the speaker actually means is “This is so stupid”); the other half (again, one expressed by female and one expressed by male) are ironic praise (PbB, i.e., negative literal meaning but positive true meaning, like “I can see you're taking it easy” said when the participant is staying up late and what the speaker actually means is “I see you're working hard”). The hearers can choose to respond either to the dictum or the implicatum or they can engage with both meanings.

The second experimental task, IPT 2, presents two BbP criticism scenarios where research subject is no longer the direct target of the ironic comment but an observer. These stimuli are presented in two conditions: (a) stereotypically male activity—two males playing football and one misses the ball and (b) stereotypically female activity—a woman being a bad driver and taking up two spots in a parking lot. The cartoons present ironic comments expressed by one of the characters toward the friend who failed: (a) “Nice skills!”—implying good agility while, actually, his agility is poor, and (b) “Nice skills!”—implying she is a good driver, while she actually is not. The two scenarios are followed by questions about the motivations and emotions of the characters. Participants also rate ironic comments on two 5-point Likert scales for their: (a) humor—funniness, the quality that makes the comment amusing and (b) malice—desire to harm others, malicious intent and ill will, as opposed to the humorous potential of a comment.

Gender stereotype self-attribution tool—two lists of personality adjectives were used as the measure of gender stereotype activation: one list consisted of 16 positive trait adjectives and the other one consisted of 16 negative trait adjectives, where 2/3 of the adjectives related to either male or female stereotype, (e.g., independent, self-confident, brave vs. caring, sensitive, emotional, etc.) and 1/3 were considered neutral (e.g., smart, creative, arrogant, conceited).

### Procedure

Participants were not told that the study specifically concerned irony. It was only explained that we were interested in knowing how people perceive and react to certain situations and comments. Participants were instructed that there were no good or bad answers. To assure study validity, male and female participants were randomly assigned to one of three experimental conditions (control, positive pretask priming, and negative pretask priming). We obtained six data sets: male control group (*n* = 44), female control group (*n* = 56), male (*n* = 32), and female (*n* = 35) groups with positive pretask priming, and male (*n* = 35) and female (*n* = 36) groups with negative pre-task priming.

Other than control, two experimental conditions were employed, where participants were conditioned by the selection of personality adjectives made available to them. The goal of the pretask conditioning was to make gender salient in order to activate gender stereotypes and induce gender stereotype-congruent inferences in the subsequent IPT. Pretask priming was based on gender self-stereotyping and the intergroup comparisons effect (Guimond et al., 2006).

In the positive pretask priming group, a social comparison paradigm was employed with a list of 16 positive trait adjectives. Participants were asked to select semantic attributes which they judged as more self-relevant when comparing themselves with outgroup members (represented by males if participants were females, or by females if participants were males). Positive trait adjectives were used with the aim of reinforcing a positive image of the self and other ingroup members at the expense of the outgroup members.

In the negative pretask priming group, a social comparison paradigm was employed with a list of 16 negative trait adjectives Participants were asked to select semantic attributes which they judged as more self-relevant when comparing themselves with out-group members. It was believed that the exposure to negative trait adjectives would provoke a negative image of the self and other ingroup members, but not outgroup members.

**Figure 1:**
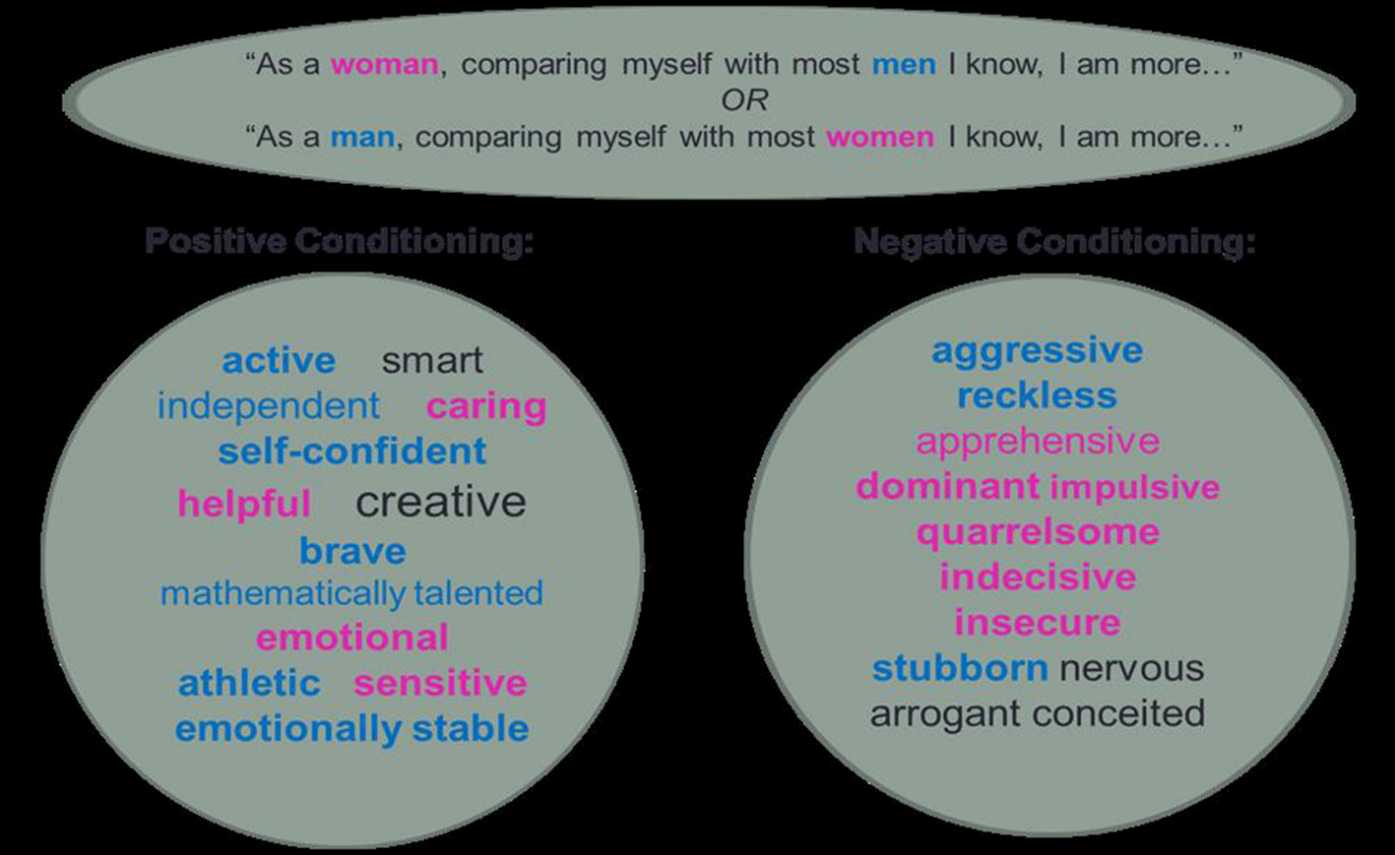
Conditioned self-attribution.

Conditioned self-attribution in positive and negative pre-task priming groups is illustrated by Figure 1.

The mention of “males” and “females” in this experimental procedure was believed to activate stereotypical knowledge of gender-relevant characteristics, which would be acknowledged as informative about the person and have an impact on the interpretation of what that person is saying. It was expected that gender category labels provided in the pretask priming would activate categorical representation and linguistic profiling.

The IPT 1 tested participants' responses to different speakers of ironic comments (ingroup or outgroup members) and to different types of irony under three different experimental conditions. The experimental design was a 2 (male vs. female) × 2 (ironic criticism vs. ironic complement) × 3 (control condition vs. positive pretask priming vs. negative pretask priming).

Where the main analyses were designed in 2 × 2 × 3 analysis of variance (ANOVA) plan, the complimentary variables were checked as unifactorial dependencies.

The data from the experiment was also used in unifactorial analyses of the relationship between (a) the level of anxiety, (b) the type of response to ironic comment (criticism and complement), (c) gender of the speaker, (d) gender of the recipient, that is, the participant (who from the role of the recipient of the ironic comment becomes herself or himself the sender of the message in either ironic or non-ironic exchange of comments).

The second experimental task, IPT 2, tested participants' perception of (a) humor and (b) malice in ironic criticism (BbP) in two different settings, involving either two ingroup members or two outgroup members. Evaluations of humor and malice were rated for each scenario on 5-point Likert scales.

In the control group, first the IPT, and then the STAI were administered to research participants. In the experimental groups, the lists of adjectives were administered first, followed by the IPT and the STAI. The order of presented tasks was kept the same for all tested individuals.

### Research objectives and hypotheses

It was hypothesized that males and females would respond differently to ironic comments coming from ingroup (same sex) or outgroup (opposite sex) members.

It was also believed that application of the pretask priming on gender stereotype activation would reinforce gender differences in attributing different meanings, congruent with these stereotypes, to ironic utterances. We hypothesized that reactions to a negative stereotype (list of negative trait adjectives), that is, a stereotype threat, would negatively impact participants' test performance (lower use of irony, lower ratings of humor, and higher ratings of malice) as opposed to the stereotype boost context, where the presentation of the list of positive trait adjectives would lead participants to improved test performance (higher use of irony, higher ratings of malice, and lower ratings of humor). It was also expected that the ironic setting (male-male vs. female–female) may determine whether relevant stereotypes are activated and it might influence the perception of irony. This is in line with Wigboldus et al. (2005) suggestion that

in an intragroup context (e.g., when females talk to females about females) a target's category membership (e.g., gender) is less likely to become salient. Consequently, stereotypic expectancies with this category are not activated, thus rendering it unlikely that linguistic biases occur. In an intergroup context, however (i.e., when either target or recipient is an outgroup member), a required category activation is more likely, and linguistic bias is expected (Beukeboom, 2014, p. 17).

We also believed that subjects with lower levels of anxiety would be more ironic in their responses to ironic comments than subjects with high levels of anxiety.

Given previous studies, it was hypothesized that males would rate the humorous potential of ironic comments higher than females. It was also hypothesized that females would rate ironic comments as more snarky and snide than males.

It was also believed that subjects with low levels of anxiety, regardless of their sex, would rate humor higher on the Likert scale.

We expected a cross-gender effect in the evaluation of humor and malice in the comments, that is, male participants giving higher rating of humor and lower rating of malice to the comment involving two outgroup members (female-female scenario) and the reverse trend in the group of female participants who would rate humor higher rather in the male-male than in the female-female scenario.

### Data analysis

Due to the exploratory nature of the study and the very distinct quality of irony in its openness to more than one interpretation, it was deemed reasonable to see what categories emerge from the collected data. Data was coded with reference to categorization by Kotthoff (2003), the classification by Clark (1996, after Hancock, 2004) and the taxonomy of irony factors and irony markers by Burgers et al. (2012). The resulting set of three categories was checked with and confirmed by two other coders. The whole multi-task IPT instrument is based on each subject's unique responses, and its reliability has been proved by high inter-rater consistency. The fourth category of “no evidence” (e.g., silence, changing the subject, no response) will not be discussed in this paper, so we are not considering it further. The two-factor analysis, based on (a) irony recognition and (b) type of response resulted in the following categories of response to irony:

1. *Ironic response* (Ir)—to what is meant: ironic intent recognized/ irony extended—acknowledgment of ironic intent and reply in terms of non-literal or equivocal language, also, but not only, with the use of ironic markers such as metaphor, hyperbole, understatement, rhetorical question, echo/repetition, question marks, emoticons, quotation marks, diminutives, laughter onomatopoeias (haha, ha ha, ahahaha, hehe, ehehehe, hohoho, ho ho);
2. *Misresponse* (Mr)—to what is said: ironic intent not recognized/ literal response—when ironic intent is missed or misinterpreted and the reply suggests that the ironic comment is not clear or taken literally (e.g., “What do you mean?,” “Really?,” “Do you really think so?”);
3. *Literal response* (Lr)—to what is meant: ironic intent recognized/ literal response—detection and correct interpretation of the ironic intent but irony is not extended and the response is literal and direct.

We also singled out *Laughter category* with all “hahahah,” ha ha ha,” emoticons, or words such as “laughter,” “smile,” “joke,” “funny,” “you must be joking,” “ha ha ha really funny” (even when implying: not funny at all) that are an expression of both humor and indignation. However, due to the fact that replies containing “laughter markers” were only a few, we did not include this category in our further analysis.

Perception of humor and malice, rated on 5-point Likert scales, were counted and analyzed separately for each experimental condition (control, positive pre-task priming, and negative pre-task priming). Also, ratings of humor and malice for each participant were analyzed with regard to their level of anxiety measured with the STAI.

## Results

Data shows that one comment can trigger completely different verbal behaviors, ranging from acknowledgment to complete disbelief or rejection.

### Reactions to ironic blame by praise vs. praise by blame by gender

The ANOVA model with mixed design was applied to analyze the results. The sex, priming, and anxiety (state and trait) were between-subject factors, while the experimental variables of sex of the interlocutor and irony type (BbP vs. PbB) were defined as within-subject variables. The occurence of three reaction types was the dependent variable, analyzed in three separate analyses.

### Blame by praise and pre-task priming effects (interaction of state, priming, and sex)

In the paradigm of ironic response to irony, the main factor was that of the interlocutor's sex, *F*_(2, 234)_ = 100.28, *p* < 0.001, η^2^ = 0.300, where males are frequent targets of ironic responses. Male subjects were also more ironic, *F*_(1, 234)_ = 5.36, *p* = 0.022, η^2^ = 0.022, but there was less irony in case of negative priming *F*_(2, 234)_ = 3.09, *p* = 0.04, η^2^ = 0.026.

**Figure 2:**
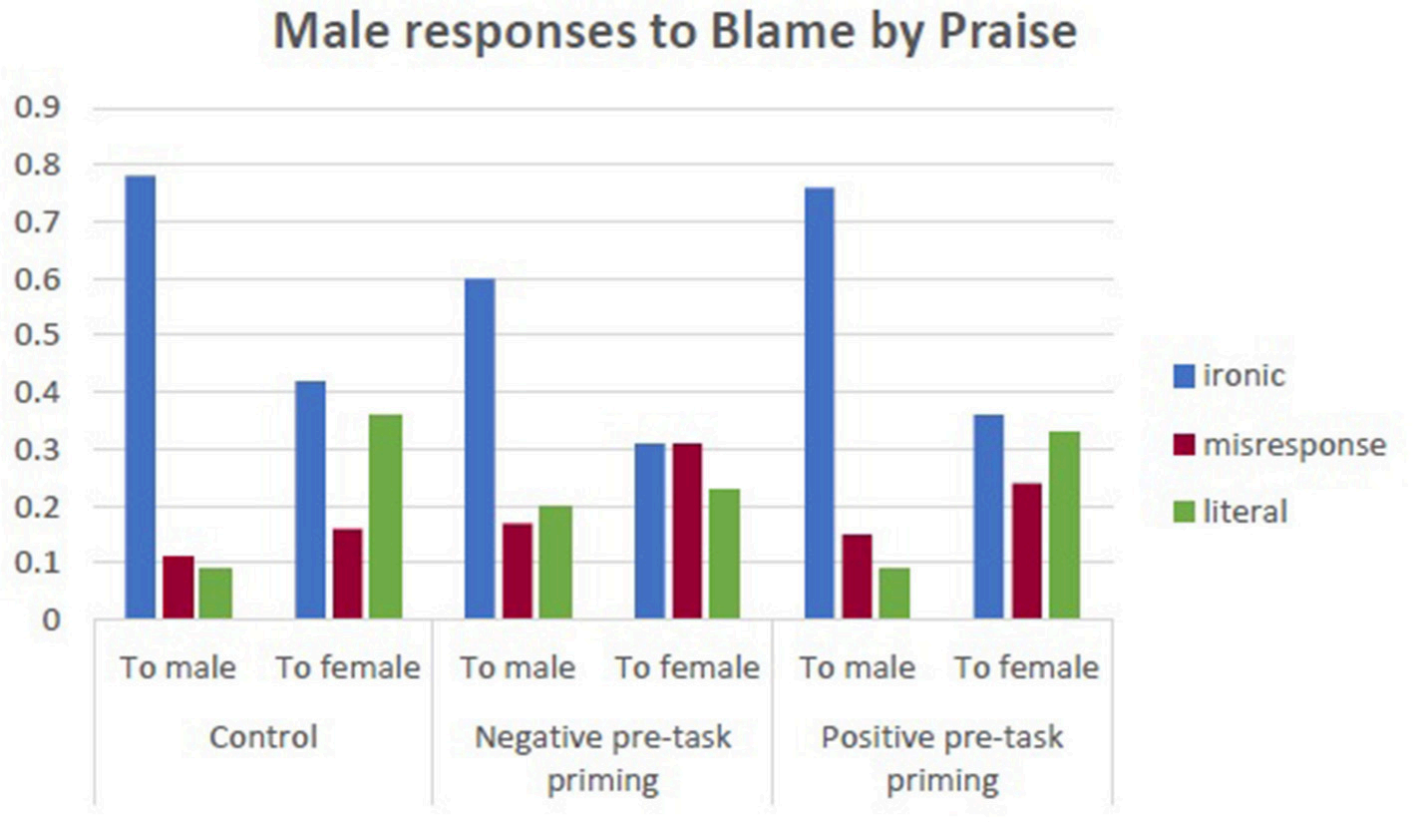
Male responses to Blame by Praise in three experimental conditions.

**Figure 3:**
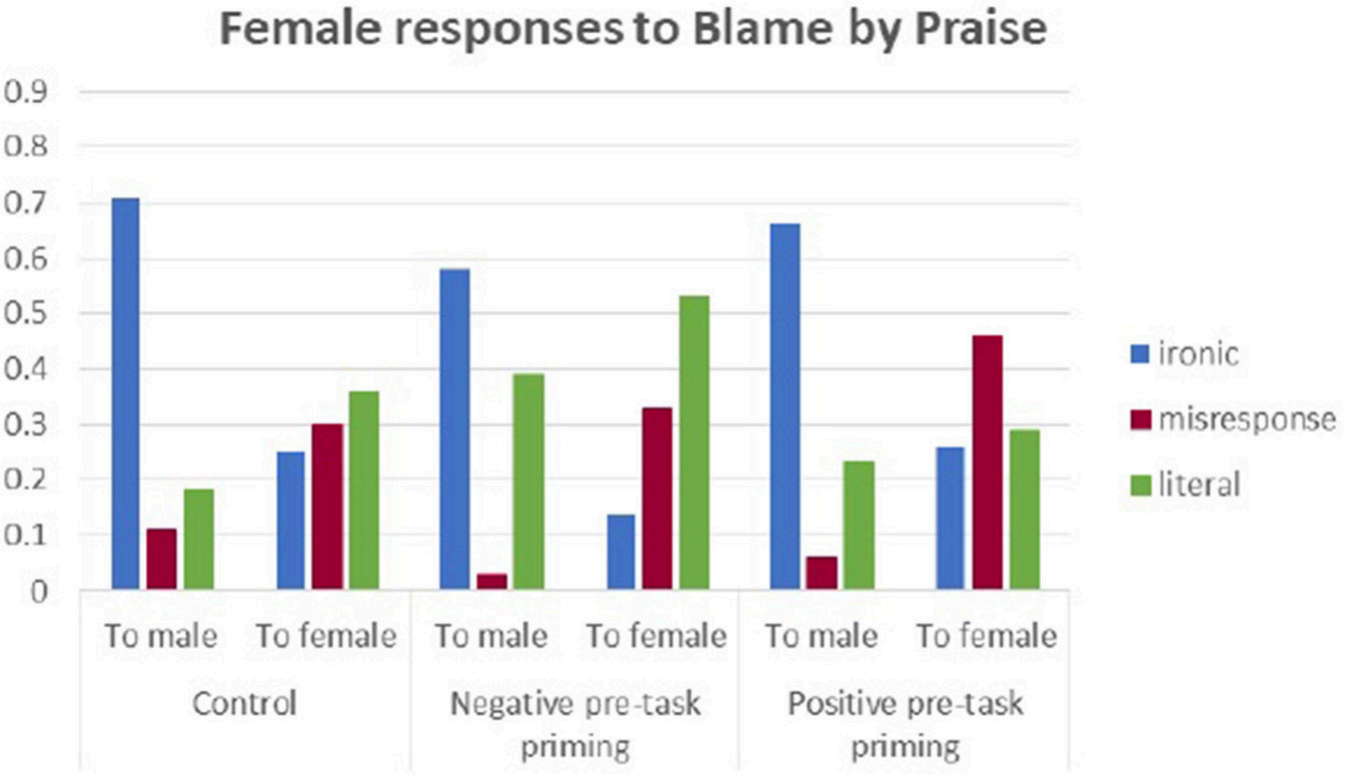
Female responses to Blame by Praise in three experimental conditions.

Figures 2, 3 illustrate the proportions of different types^1^ of response to BbP in the three experimental conditions.

**Figure 4:**
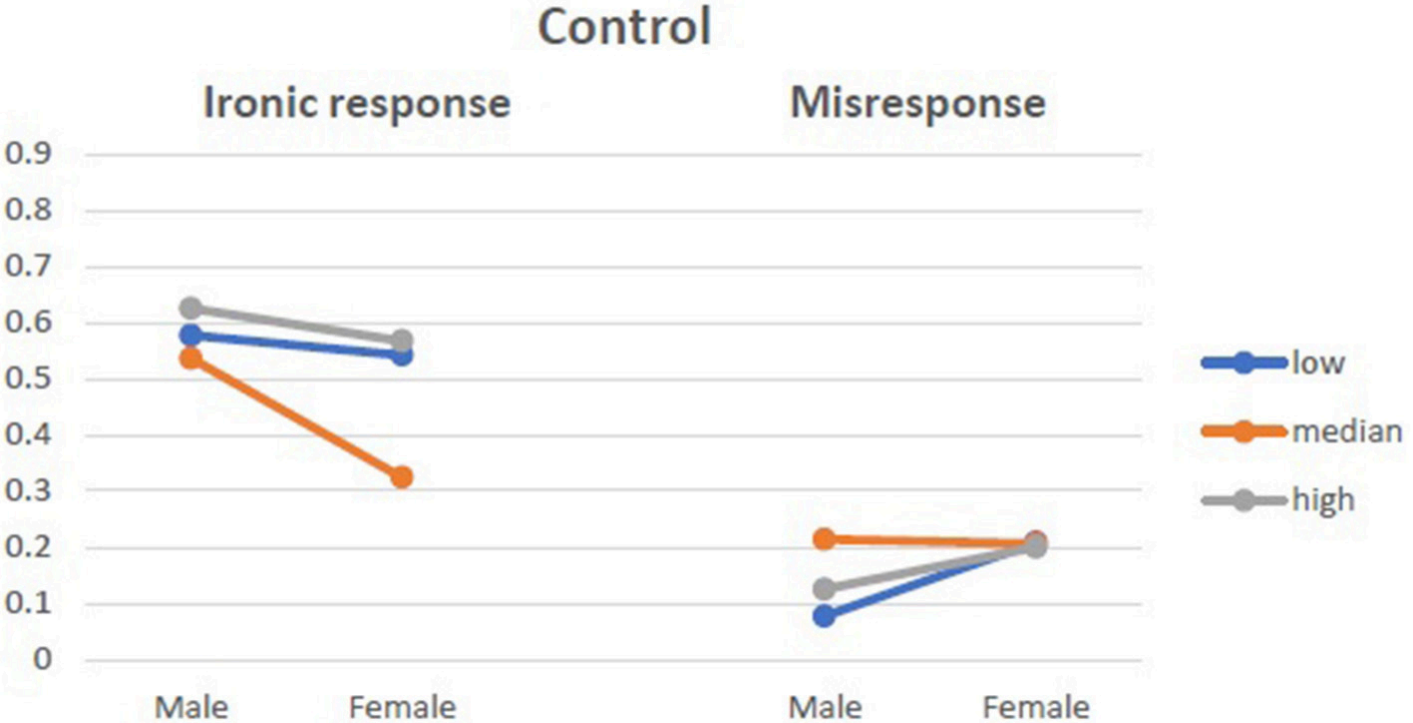
Ironic response vs. Misresponse to Blame by Praise in relation to state anxiety in control group.

**Figure 5:**
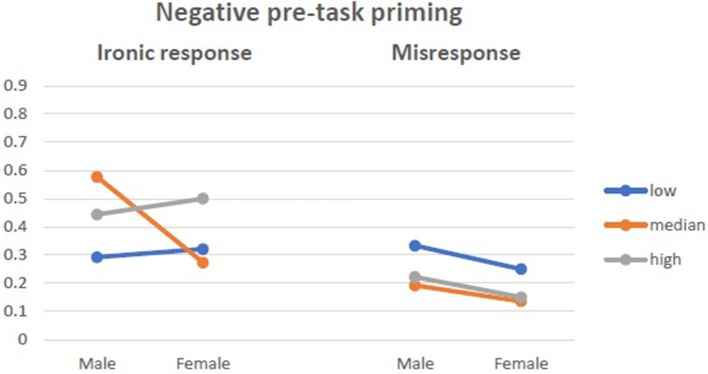
Ironic response vs. Misresponse to Blame by Praise in relation to state anxiety in negative pre-task priming.

**Figure 6:**
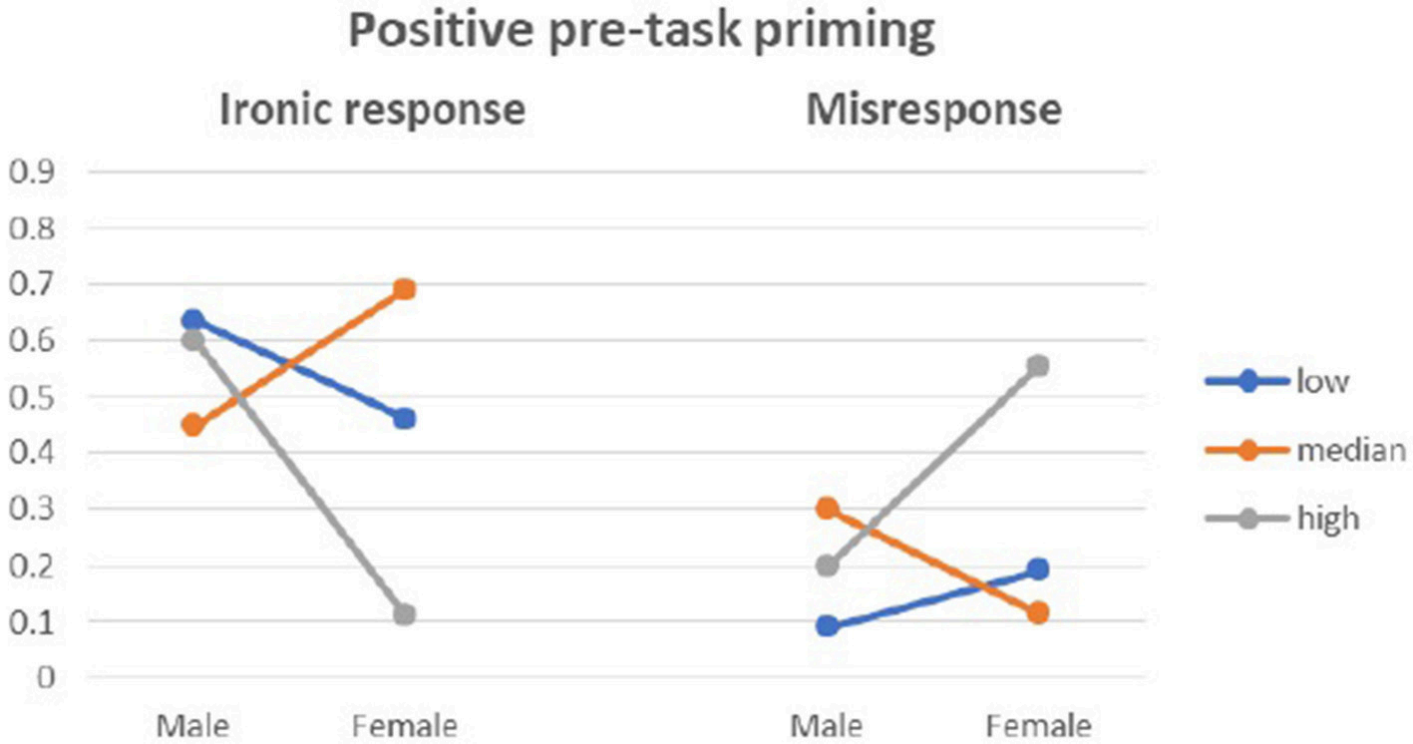
Ironic response vs. Misresponse to Blame by Praise in relation to state anxiety in positive pre-task priming.

There was an interactive effect of state anxiety moderating the above dependencies, *F*_(4, 234)_ = 4.444, *p* = 0.002, η^2^ = 0.077. Anxiety as a trait did not moderate the above effects. Women with median anxiety were surprisingly more ironic in the condition of positive pretask priming, while in the two other groups, they tended not to be ironic in their responses. The proportions of ironic responses and misresponses from women and men with different levels of state anxiety are presented in Figures 4–6.

Misresponse to irony depended on “who is speaking to whom.” Significant was the sex of the interlocutor, *F*_(1, 234)_ = 35.09, *p* < 0.001, η^2^ = 0.13, but more interesting was the interaction between the sex of the respondent and his/her interlocutor, *F*_(1, 234)_ = 9.80, *p* = 0.002, η^2^ = 0.04. Males reacted with misresponse in an almost similar way to different interlocutors, while female subjects were significantly more likely to react with misresponse to a female interlocutor.

In a supplementary analysis, we also observed that high-anxious women mostly misresponded in the positive priming condition.

Anxiety as a trait did not moderate the above effects.

In literal responses, the main effect of the interlocutor was observed, *F*_(1, 234)_ = 15.63, *p* < 0.001, η^2^ = 0.063, as well as the effect of the subject's sex, *F*_(1, 234)_ = 7.10, *p* = 0.008, η^2^ = 0.029. No higher interactions were present.

No effects of state or trait anxiety were observed.

### Praise by blame and pre-task priming effects (interaction of state, priming, and sex)

The analysis of the expected interaction between the priming effect, subject's sex, and interlocutor's sex in ironic response to irony was not significant, *F*_(2, 234)_ = 2.25, *p* > 0.1, η^2^ = 0.02. However, the main effect of the subject's sex was significant, *F*_(2, 234)_ = 12.31, *p* < 0.001 η^2^ = 0.05, just as the effect of the interlocutor's sex, *F*_(2, 234)_ = 7.31, *p* = 0.007, η^2^ = 0.03, but they were additive. Irony was directed more frequently at women, independently of who the speaker was. Males turned out to respond more with ironic responses (to both men and women). Priming had no direct or interactive effects in this case. Supplementary analyses did not confirm any interactions with state and trait anxiety.

**Figure 7:**
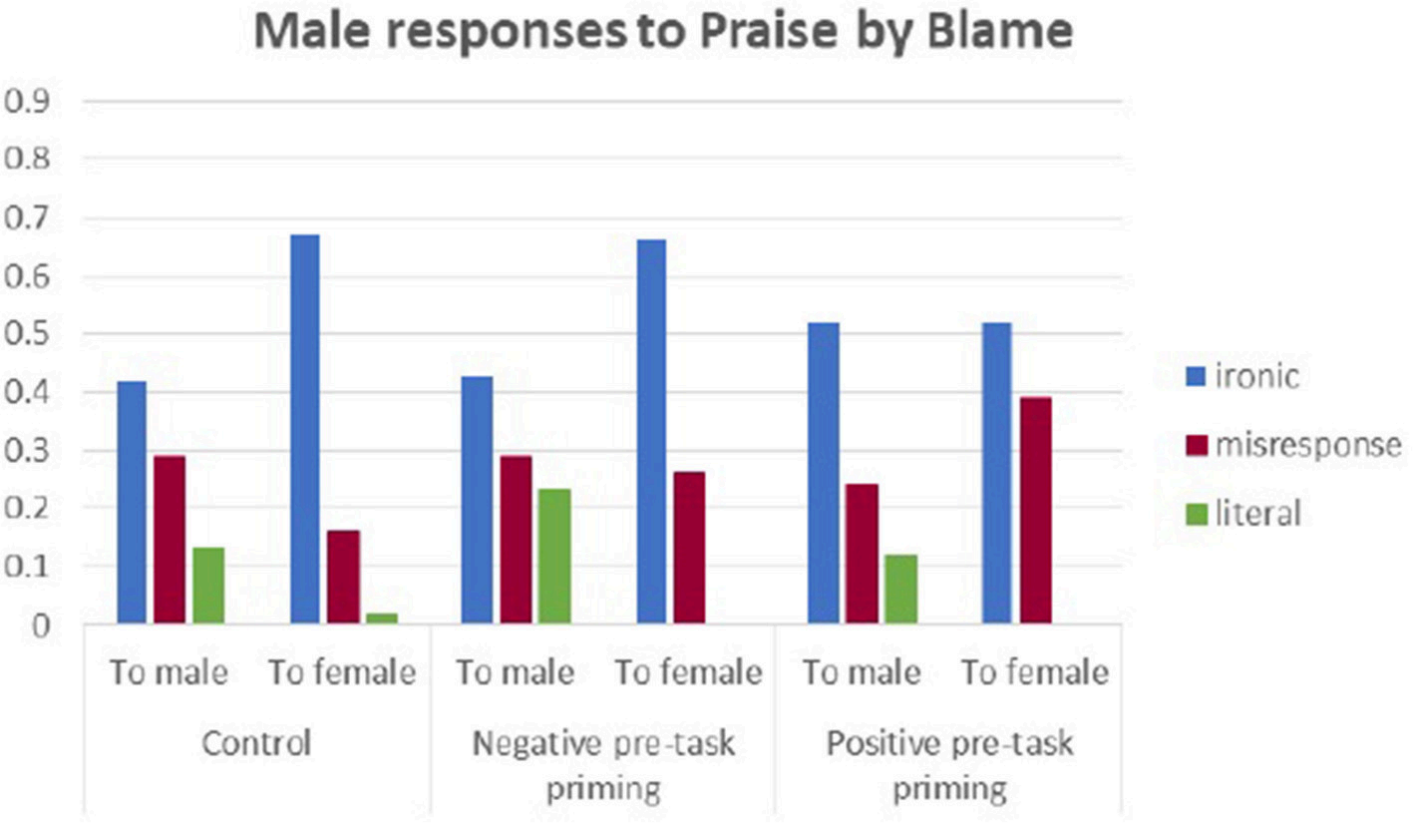
Male responses to Praise by Blame in three experimental conditions.

**Figure 8:**
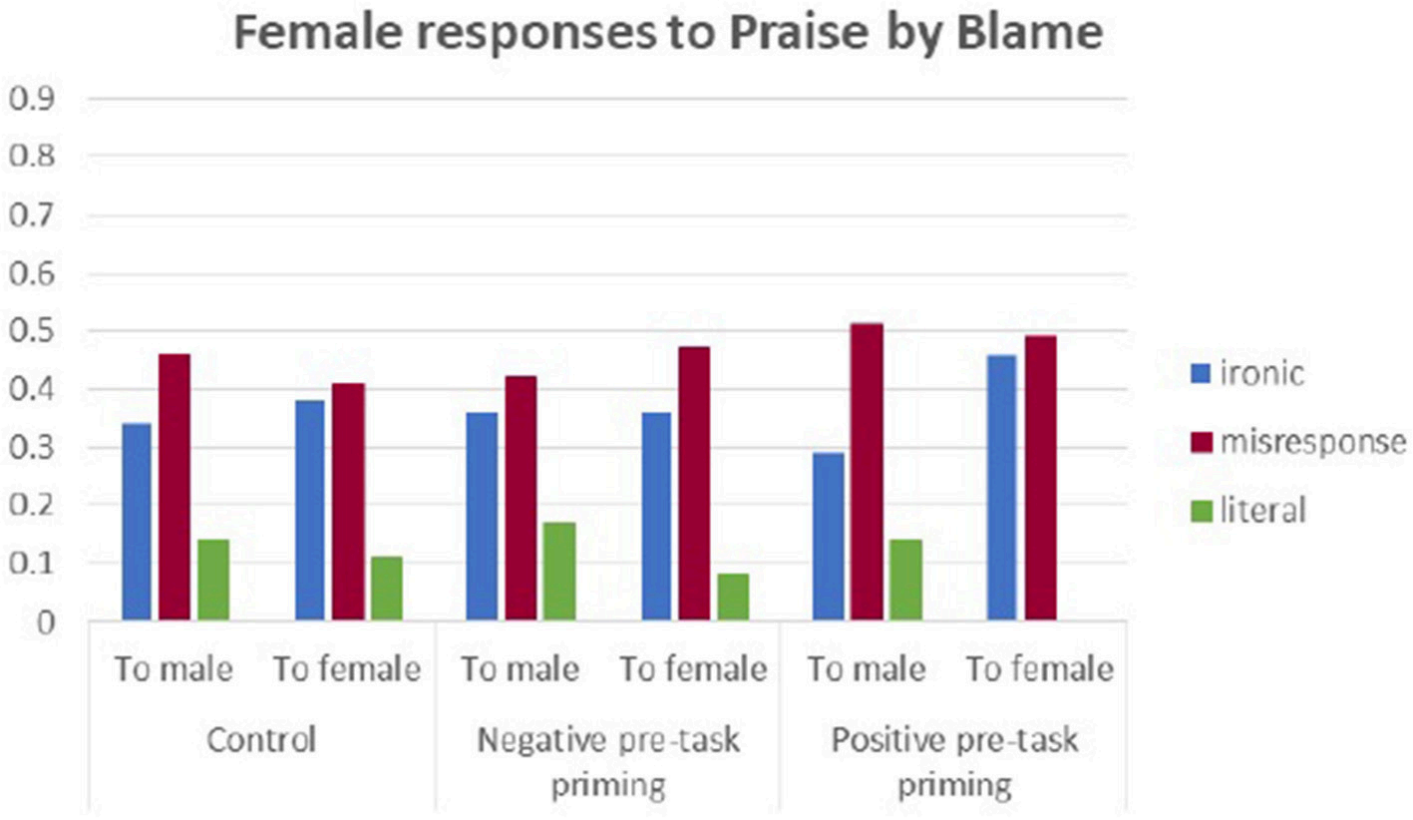
Female responses to Praise by Blame in three experimental conditions.

Figures 7, 8 illustrate the proportions of different types^2^ of response to PbB in the three experimental conditions.

Misresponse to irony was significantly more frequent in female than in male subjects. Neverthless, the expected interaction (priming × subject's sex × interlocutor's sex) was not confirmed, *F*_(2, 234)_ = 1.01, *p* > 0.1, η^2^ = 0.01. No other main or interactive effects were confirmed. No interaction with state anxiety was observed, only a weak non-linear interaction of trait anxiety with the subject‘s sex and the interlocutor’s sex was significant, *F*_(2, 208)_ = 3.25, *p* = 0.044, η^2^ = 0.03. Higher levels of misresponse probability were observed in low-anxiety males and medium-anxiety females when reacting to irony coming from male.

Literal responses were much more probable toward male than female interlocutor, but no expected interactions of sex, interlocutor, and priming was observed, *F*_(2, 208)_ = 3.25, *p* = 0.044, η^2^ = 0.03, so the effect was not moderated by sex and priming. No anxiety influence or interactions were found.

### Comparative analysis of ironic setting and perception of humor and malice in irony

Perceptions of humor and malice in the male-male situation vs. female-female situation were analyzed with the dependent-samples *t*-test (Supplementary Table 1), performed in six groups (for men and women in the three experimental conditions).

**Figure 9:**
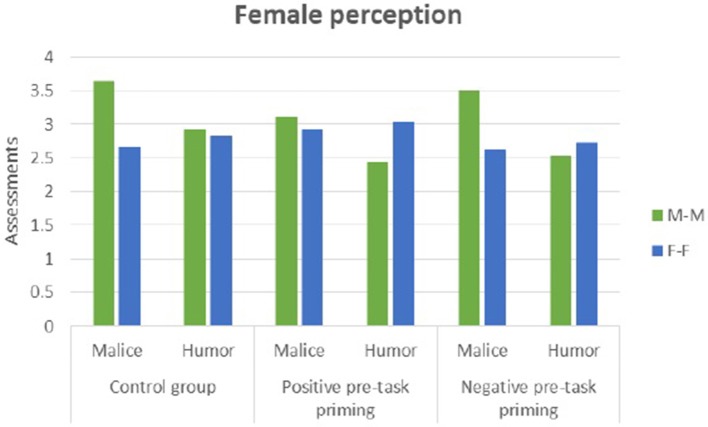
Male perception of humor and malice in male-male (M-M) and female-female (F-F) dyads.

**Figure 10:**
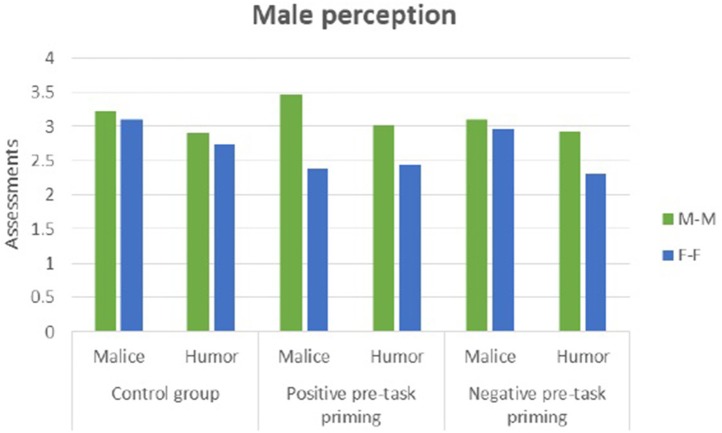
Female perception of humor and malice in male-male (M-M) and female-female (F-F) dyads.

In the positive condition, men (see Figure 9) perceived irony in the male-male setting as more malicious (*t* = 3.20, *p* = 0.003) than in the female-female setting. In the control and negative conditions, women (see Figure 10) perceived irony in the male-male setting as more malicious than in the female-female setting (*t* = 3.97, *p* = 0.000 in the control and *t* = 2.77, *p* = 0.009 in the negative condition, respectively).

No differences in ironic humor perception between male-male and female-female settings were found (Supplementary Datasheet 1).

### Anxiety and irony perception by male and female subjects in the three experimental conditions

When investigating the relationship between state/trait anxiety and irony perception, we computed the Kendall-s Tau correlations between the standardized STAI scores and two scales assessing subjective perception of ironic statements (they were assessed independently in the aspects of malice and humor). The coefficients have been computed in six groups (Supplementary Table 2): for male and female subjects, according to the three experimental conditions.

In the control conditions, males with higher state anxiety perceived ironic statements made by women as more humorous (*tau* = 0.251, *p* = 0.028), and statements made by men as less humorous (*tau* = −0.245, *p* = 0.030).

In the negatively primed group, the second effect was similar: high state-anxious men perceived more humor (*tau* = 0.239, *p* = 0.047) in women's ironic statements. In the positive condition, men with higher state and trait anxiety perceived women's ironic statements as less malicious (respectively *tau* = −0.252, *p* = 0.041, and *tau* = −0.238, *p* < 0.048), and high trait-anxiety men assessed men's ironic statements as funnier and more humorous (*tau* = 0.263, *p* < 0.031).

In the control and negative conditions, women presented no significant correlation between anxiety and irony perception. In the positively primed group, women with high trait anxiety perceived men's ironic statements as slightly more malicious (*tau* = 0.238, *p* = 0.047) but they perceived women's ironic statements as definitely less malicious (the strongest correlation in the study: *tau* = −0.461, *p* = 0.001).

## Discussion

### Gender effect in irony distribution

The exploratory data analysis of reactions to irony showed a relationship between gender and response to irony, in other words, between who says what to whom, which we call the *outlook effect* (the mental attitude which refers to the gender bias in use of verbal irony in communication, where the sender modifies the content of the message by its recipient, just as in personal email correspondence carrier). In five out of six male experimental groups, most participants reacted to irony with irony, in both BbP and PbB contexts, regardless of whether they spoke to another male or female. Ironic comments simply triggered mostly ironic replies in men. The results of our research are also in line with Holmes' claim that “women and men develop different patterns of language use” (Holmes, 1998, p. 462). Ironic response was significantly more frequent as a reply to males than to females. In the negative pretask priming group, ironic responses and misresponses became equally frequent categories of the reply to an ironic BbP comment from female.

While men turned out to be pretty stable in how they replied to irony, in all experimental conditions, regardless of the recipient's gender, female participants proved to be more unpredictable in their reactions. An ironic response (Ir) from a female to an ironic BbP comment coming from a male (the opposite sex, i.e., an outgroup member) showed to be the most frequent category in all the experimental conditions. This pattern of Ir > Lr> Mr was broken by the change in the sex of the interlocutor and by the shift to the PbB context. The most frequent category of reaction to BbP comment made by females was literal response (Lr, for thecontrol group and the positive pretask priming group). Misresponse (Mr, where criticism was mistaken for comfort and reassurance or when the superficial level of praise was genuinely acknowledged) was the most frequent category in the negative pretask priming female group.

In the PbB context, women replied most frequently with misresponse, regardless of who they spoke to (another female or a male) and this pattern was kept in all six experimental conditions. Ironic replies to another female were also quite frequent, while literal replies were rare and used more toward outgroup (male) members.

In the PbB context, men were more ironic in their responses to females than males. They still kept the significantly high frequency of ironic responses, but the proportion of use was reversed when compared with the BbP context, where they were more ironic toward same-sex interlocutors (i.e., another male).

We can see that two types of contextual frames constructed to convey (a) a desirable situation calling for a praise (a positive context condition) and (b) an undesirable situation endorsing criticism (a negative context condition), provoked different types of reactions.

In the BbP context, females used more irony to males but not to another female. In the PbB context, they reacted mostly with misresponses to both the ingroup and outgroup members. Additionally, literal responses were given more frequently to males (outgroup members) than females (ingroup members). However, male participants were more ironic to males in the BbP context, but to females—in the PbB context. Also, only a few literal comments were used with females and significantly more were used with males in the PbB context.

Women are more changeable, or rather, flexible, and more likely to adapt their behavior to circumstances than men. There are many factors that may explain some of the differences in the results, such as the greater risk aversion of women (Dwyer et al., 2002; Fletschner et al., 2010) or varied social distance between participants. However, we believe that the gender effect in irony distribution can also be attributable, at least to a certain extent, to a greater context sensitivity of women (Gilligan, 1982; Cadsby and Maynes, 1998). Gilligan (1982) claimed that women act more in terms of care orientation and cooperation toward other people while men act more in terms of abstract justice, rights, and obligations. Faced with a moral dilemma, that is, a choice to make, an individual with a care perspective will consider it in a contextualized fashion that takes into account how the individual is related to others who are involved in the dilemma. As a consequence, women may display different behavior in different contexts as a function of the contextualized features involved while men will tend to display behavior that is less context-sensitive and more rule-based. Another way of interpreting gender differences in use of irony can be in line with the reinterpretation of Cadsby and Maynes' (1998) view that women are more likely to follow conditional, as opposed to unconditional, rules—ones that depend on the specific context at hand.

The explanation of these differences can also lie in the theory of sexual selection. Females like predictability in their mates as it allows them to make good long-term decisions, and to deal with changing circumstances if they know their male is consistent (Schuett et al., 2010).

Could it be that even subtleties in our linguistic behavior reflect the true nature of our species? The study led by Schuett et al. (2010) shows that in most species, males show more consistent, predictable behaviors, particularly in relation to parental care, aggression, and risk-taking. Also, men are more inclined to engage in high-risk activities (Howland et al., 1996; Byrnes et al., 1999), of which irony can be an example on a symbolic level, like Colston and Lee (2004) suggest.

The results of Experiment 1 have also presented evidence supporting the activation of the mechanism of linguistic intergroup bias (Maass et al., 1989; Wigboldus and Douglas, 2007) in non-literal communication. The experiment shows that toward one's own group, that is, toward other women, irony was not frequently used in the context of ironic BbP, (covert criticism). Literal responses were significantly more present in responses to irony coming from an ingroup member in the negative pretask priming group. However, irony was kept in responses to ironic criticism expressed toward outgroup members, where literal comments were significantly less frequent.

Females used irony more often toward males (outgroup members) than to females (ingroup members), but only in the BbP context. However, they used more irony to ingroup members in the PbB (covert complement) than in the BbP (covert criticism) context and this trend was reversed when they used irony toward outgroup members, that is, males.

Male participants in the PbB (covert complement) context were more ironic toward females than males and used more literal responses when addressing the ingroup members, that is, other males.

These results also corroborate the results of the study by Milanowicz and Bokus (2013), which revealed that a simple change of the interactional setting, that is, of who is speaking to whom shifts the perception of the interlocutor and altogether modifies the process and the result of moral reasoning and communication.

### Anxiety and responses to irony

The moderating effect of state anxiety on verbal reactions to ironic comments reflects the fact that irony is not a general quality of a person, but rather a state. Irony is unique depending on the context, and we do not respond to it by calling upon a database of jokes. Irony is being made again and anew, in the “here and now.” Irony is more of a property of communication than of individual subjects. On the other hand, some people think of themselves as having more ironic tendencies than others. Our research draws attention to the fact that the amount of irony can be modified by conditioning. However, we do not rule out the role of other individual differences in the perception of irony and production of ironic remarks.

The perceived degree of lightheartedness or malice in ironic comments was previously linked to gender differences. Milanowicz (2013) showed that females displayed a more negative attitude to ironic comments than men, and it was hypothesized as being linked to different anxiety levels. To this end, IPT results were correlated with scores on the STAI. Anxiety proved to be a moderate predictor of irony comprehension and the willingness to use irony.

### Is irony a funny thing?

An unquestionable asset of this study is that it compares irony across different communicative situations within one modality: as if a real situation presented in a classic, written form. Unlike in Hancock's (2004) research paradigm, modality is kept constant and the study participants, that is, ironic speakers-to-be, can produce irony with the same set of linguistic tools. In lieu of intonation, facial expression, or gestures, they made good use of typographic cues and rhetorical figures,

We explored perception and use of irony by its direct “targets” or “recipients” (Experiment 1) as well as by its “witnesses” (Experiment 2). It is quite surprising that the self-attribution conditioning impacted male and female perception of malice and humor in a reversed way. It is also interesting that the differences are shown only in the assessment of male-male ironic setting. This might be the result of the activation of different mental representations associated with the social categories of men and women. For example, the label “woman” activates a different stereotype than the label “man.” It also seems, based on these research results that irony acts as a filter, and its regulatory mechanism works different for men and different for women. It may relate to the fact that stereotypically, men have high power/status but low acceptance for expressing emotions. Women have high acceptance for expressing (negative) emotions but lower status, so irony works for men as a euphemism (for what cannot be said openly) and as loaded language for women. If ratings and evaluations of the same situation vary as to the context, mood, and perception of its evaluator, the above demonstrates that stereotypic expectancies are flexible and can be overridden under certain conditions.

Irony is omnipresent. Just take a look into private and public space to see how almost every category of contemporary reality—relationships, advertising, politics, television, or social networks—use and misuse this concept. Understanding irony (which is believed to be accessible only to humans) is maybe the closest experience we can have to mind reading. AI researchers are venturing into cracking irony, IT specialist are taking interest in linguistic profiling and HR managers are still probably confused as to whether they were funny or just mean. We are used to taking a binary approach to many things we try to understand, and we take that approach to irony as well. Something is ironic, that is, funny or something is not ironic, that is, not funny. However, we are quite certain that this binary character will soon be transgressed, as we will need an updated approach to understanding the trans-gender and trans-genetic world of the future, where the binary system of two definite states (0 or 1) will be just an illusion.

## Conclusions

Irony is not a trifling matter. It is a social tool, which can make or break when it comes to having a mutual understanding and building rapport. We assumed that a higher level of anxiety in adults could account for the misperception of ambiguous comments as threatening and provoke tuning out of rather than tuning into an ironic exchange of comments. This hypothesis was not confirmed.

We showed, however, that linguistic bias is present in the use of irony, and we proposed that it might result from the gender effect and essentialist beliefs about social categories.

Taking into account individual preferences, variable across individuals while stable within individuals, can allow for more refined theoretical models of verbal irony as well as better predictions of communicative choices. More research and more data might lead to further discovery of patterns and mechanisms that impact the quality of interactions in the real-life context.

Our present study is a step forward to clarify the origins of individual differences in the perception of irony, its social functions and both, bright and dark sides of humor.

We plan in the future to explore the capacity to distinguish between the positive and negative functions of irony under the influence of gelotophobic fear, that is, the fear of laughter identified with ridicule (Ruch and Proyer, 2008a,b). We assume that gelotophobes will perceive positive irony as negative (as targeting their appearance, undermining their competence or achievements, etc.), more often than individuals with no fear of being laughed at.

## Limitations

Emerging adults (i.e., adults between 18 and 25 years of age) constitute the majority of our research subjects—with mean age of 23.92 years for the total sample they form an in-between group, having completed adolescence but not yet entered adulthood. This is a stage of cognitive transition and recognizing different perspectives (Arnett, 2006).

We tried to control for the same advanced level in cognition by having a homogenous group of people with a university-level education (completed or in progress). We analyzed data collected from 238 subjects divided into six different experimental groups. The sample size of the experimental groups may pose risk of Type II errors.

All our subjects were white, European, native Polish speakers, living in Warsaw, (the capital city—an economically developed area) Poland (almost 90% Roman Catholic), which makes us think that a more diversified ethnic background would call for cross-cultural research. For example, Irish/British citizens consider themselves highly ironic. Maybe it has something to do with the puritan Victorian society, where one just could not speak up about certain (embarrassing) things. How much has irony to do with taboo or religion and social beliefs?

And lastly, the very wording of the ironic comment (type of comment) can also provoke a given type of response on its own and act as a moderator, which is uncontrolled for.

## Author contributions

The research reported in this article is part of AM's doctoral dissertation at the Faculty of Psychology of the University of Warsaw, Poland, supervised by the third author. AM: Data collection, data analysis, drafting manuscript. AT: Data analysis. BB: data collection, data analysis, revision of manuscript.

### Conflict of interest statement

The authors declare that the research was conducted in the absence of any commercial or financial relationships that could be construed as a potential conflict of interest.

## Footnotes

^1^The participants' responses to ironic Blame by Praise were classified on the basis of ratings from three independent judges into three categories (*ironic, misresponse, literal*). The inter-rater consistency was measured with Kendall's coefficient; *W* = 0.987 for male responses and *W* = 0.993 for female responses. Kendalls' *W* was calculated from data obtained from 111 men and 127 women.

^2^The participants' responses to ironic Praise by Blame were classified on the basis of ratings from three independent judges, into three categories (*ironic, misresponse, literal*). The inter-rater consistency was measured with Kendall's coefficient; *W* = 0.988 for male responses and *W* = 0.984 for female responses. Kendalls' *W* was calculated from data obtained from 111 men and 127 women.
