# Supplementary material for: When Sugar-Coated Words Taste Dry: The Relationship between Gender, Anxiety, and Response to Irony
Source: Front Psychol. 2017 Dec 19;8:2215. doi: 10.3389/fpsyg.2017.02215 (PMC5742492; doi:10.3389/fpsyg.2017.02215)
Supplement: Supplementary file 1 [file Table1.pdf]

Table 1. Results of the dependent-samples *t*-test.

Sex		mean	N	Std dev	*t*	df	significance
Male Control	Malice men	3,21	43	1,19	0,39	42	0,698
	Malice women	3,09	43	1,32			
	Humor men	2,90	42	1,32	0,53	41	0,596
	Humor women	2,74	42	1,34			
Female Control	Malice men	3,63	56	1,00	3,97	55	0,000
	Malice women	2,66	56	1,05			
	Humor men	2,91	56	1,20	0,32	55	0,750
	Humor women	2,82	56	1,11			
Male Negative	Malice men	3,09	33	1,23	0,34	32	0,737
	Malice women	2,97	33	1,31			
	Humor men	2,91	32	1,35	1,49	31	0,147
	Humor women	2,31	32	1,28			
Female Negative	Malice men	3,50	36	1,06	2,77	35	0,009
	Malice women	2,61	36	1,15			
	Humor men	2,53	36	1,08	-0,63	35	0,535
	Humor women	2,72	36	1,21			
Male Positive	Malice men	3,45	33	1,15	3,20	32	0,003
	Malice women	2,39	33	1,25			
	Humor men	3,00	33	1,25	1,64	32	0,110
	Humor women	2,45	33	1,18			
Female Positive	Malice men	3,11	35	1,16	0,57	34	0,571
	Malice women	2,91	35	1,31			
