# Supplementary material for: When Sugar-Coated Words Taste Dry: The Relationship between Gender, Anxiety, and Response to Irony
Source: Front Psychol. 2017 Dec 19;8:2215. doi: 10.3389/fpsyg.2017.02215 (PMC5742492; doi:10.3389/fpsyg.2017.02215)
Supplement: Supplementary file 2 [file Table2.pdf]

Table 2. Kendall's Tau Correlation Coefficients.

Priming	Sex				X-1 (state)	X-2 (trait)
control	Male	Kendall Tau b	Malice-Male	Correlation coefficient	-.101	.017
				Sig. (1-tailed)	.217	.447
				N	38	38
			Malice-Female	Correlation coefficient	.028	- .158
				Sig. (1-tailed)	.412	.112
				N	38	38
			Humor- Male	Correlation coefficient	-.245*	-.063
				Sig. (1-tailed)	.030	.316
				N	37	37
			Humor-Female	Correlation coefficient	.251*	.187
				Sig. (1-tailed)	.028	.079
				N	37	37
	Female	Kendall Tau b	Malice-Male	Correlation coefficient	.057	.064
				Sig. (1-tailed)	.301	.281
				N	56	55
			Malice-Female	Correlation coefficient	-.105	-.060
				Sig. (1-tailed)	.165	.292
				N	56	55
			Humor - Male	Correlation coefficient	.012	.060
				Sig. (1-tailed)	.453	.290
				N	56	55
			Humor - female	Correlation coefficient	.058	.016
				Sig. (1-tailed)	.294	.442
				N	56	55
Negative	Male	Kendall Tau b	Malice-Male	Correlation coefficient	-.179	-.026
				Sig. (1-tailed)	.098	.426
				N	33	32
			Malice-Female	Correlation coefficient	.206	.138
				Sig. (1-tailed)	.067	.163
				N	33	32
			Humor - Male	Correlation coefficient	,000	,020
				Sig. (1-tailed)	,500	,444
				N	32	31
			Humor-Female	Correlation coefficient	.239*	.195
				Sig. (1-tailed)	.047	.091
				N	32	31
	Female	Kendall Tau b	Malice-Male	Correlation coefficient	.140	.172
				Sig. (1-tailed)	.153	.102
				N	35	35
			Malice-Female	Correlation coefficient	.034	.036
				Sig. (1-tailed)	.400	.395
				N	35	35

			Humor - Male	Correlation coefficient	-,114	-,171
				Sig. (1-tailed)	,203	,104
				N	35	35
			Humor - female	Correlation coefficient	,187	,063
				Sig. (1-tailed)	,086	,320
				N	35	35
Positive	Male	Kendall Tau b	Malice-Male	Correlation coefficient	.067	-.075
				Sig. (1-tailed)	.322	.300
				N	31	32
			Malice-Female	Correlation coefficient	-.252*	-.238*
				Sig. (1-tailed)	.041	.048
				N	31	32
			Humor - Male	Correlation coefficient	.013	.263*
				Sig. (1-tailed)	.465	.031
				N	31	32
			Humor - Female	Correlation coefficient	-.199	-.223
				Sig. (1-tailed)	.085	.059
				N	31	32
	Female	Kendall Tau b	Malice-Male	Correlation coefficient	.058	.238*
				Sig. (1-tailed)	.336	.047
				N	35	32
			Malice-Female	Correlation coefficient	-.209	-.461**
				Sig. (1-tailed)	.062	.001
				N	35	32
			Humor - Male	Correlation coefficient	-.048	.075
				Sig. (1-tailed)	.364	.301
				N	35	32
			Humor - Female	Correlation coefficient	.152	.002
				Sig. (1-tailed)	.132	.493
				N	35	32

* p<.05 (1-tailed)
** p<.001(1-tailed)
