# Supplementary material for: When Sugar-Coated Words Taste Dry: The Relationship between Gender, Anxiety, and Response to Irony
Source: Front Psychol. 2017 Dec 19;8:2215. doi: 10.3389/fpsyg.2017.02215 (PMC5742492; doi:10.3389/fpsyg.2017.02215)
Supplement: Supplementary file 3 [file DataSheet1.pdf]

Datasheet 1. Comparative analysis of ironic setting and humor/malice perception.

Priming	Variable	Sex of Observer	N	Mean	Std. dev.	t	df	significance
Control	M-M Malice	Male	43	3,21	1,19	-1,89	97	0,062
		Female	56	3,63	1,00			
	F-F Malice	Male	43	3,09	1,32	1,81	97	0,073
		Female	56	2,66	1,05			
	M-M Humor	Male	42	2,90	1,32	-0,02	96	0,981
		Female	56	2,91	1,20			
	F-F Humor	Male	42	2,74	1,34	-0,34	96	0,738
		Female	56	2,82	1,11			
Negative	M-M Malice	Male	33	3,09	1,23	-1,48	67	0,143
		Female	36	3,50	1,06			
	F-F Malice	Male	34	3,03	1,34	1,40	68	0,165
		Female	36	2,61	1,15			
	M-M Humor	Male	32	2,91	1,35	1,28	66	0,205
		Female	36	2,53	1,08			
	F-F Humor	Male	33	2,39	1,34	-1,07	67	0,29
		Female	36	2,72	1,21			
Positive	M-M Malice	Male	33	3,45	1,15	1,22	66	0,228
		Female	35	3,11	1,16			
	F-F Malice	Male	33	2,39	1,25	-1,67	66	0,099
		Female	35	2,91	1,31			
	M-M Humor	Male	33	3,00	1,25	1,99	66	0,051
		Female	35	2,43	1,12			
	F-F Humor	Male	33	2,45	1,18	-1,84	66	0,07
		Female	35	3,03	1,38			

Sex of Observer		mean	N	Std. dev.	t	df	significance
Male Control	M-M Malice	3,21	43	1,19	0,39	42	0,698
	F-F Malice	3,09	43	1,32			
	M-M Humor	2,90	42	1,32	0,53	41	0,596
	F-F Humor	2,74	42	1,34			
Female Control	M-M Malice	3,63	56	1,00	3,97	55	0,000
	F-F Malice	2,66	56	1,05			
	M-M Humor	2,91	56	1,20	0,32	55	0,750
	F-F Humor	2,82	56	1,11			
Male Negative	M-M Malice	3,09	33	1,23	0,34	32	0,737
	F-F Malice	2,97	33	1,31			
	M-M Humor	2,91	32	1,35	1,49	31	0,147
	F-F Humor	2,31	32	1,28			
Female Negative	M-M Malice	3,50	36	1,06	2,77	35	0,009
	F-F Malice	2,61	36	1,15			
	M-M Hum	2,53	36	1,08	-0,63	35	0,535
	F-F Hum	2,72	36	1,21			
Male Positive	M-M Malice	3,45	33	1,15	3,20	32	0,003
	F-F Malice	2,39	33	1,25			
	M-M Humor	3,00	33	1,25	1,64	32	0,110
	F-F Humor	2,45	33	1,18			
Female Positive	M-M Malice	3,11	35	1,16	0,57	34	0,571
	F-F Malice	2,91	35	1,31			
	M-M Humor	2,43	35	1,12	-1,71	34	0,096
	F-F Humor	3,03	35	1,38			
